# Comparison of Efficacy and Safety Between Low-Dose Ziprasidone in Combination With Sertraline and Ziprasidone Monotherapy for Treatment-Resistant Patients With Acute Exacerbation Schizophrenia: A Randomized Controlled Trial

**DOI:** 10.3389/fphar.2022.863588

**Published:** 2022-04-26

**Authors:** Hui Shi, Jing Xu, Xiaoe Lang, Hanjing Emily Wu, Mei Hong Xiu, Xiang Yang Zhang

**Affiliations:** ^1^ Department of Psychiatry, Beijing Chao-Yang Hospital, Capital Medical University, Beijing, China; ^2^ Qingdao Mental Health Center, Qingdao Medical University, Qingdao, China; ^3^ Department of Psychiatry, Shanxii Medical University, Taiyuan, China; ^4^ Department of Psychiatry and Behavioral Sciences, The University of Texas Health Science Center at Houston, Houston, TX, United States; ^5^ Peking University HuiLongGuan Clinical Medical School, Beijing HuiLongGuan Hospital, Beijing, China; ^6^ CAS Key Laboratory of Mental Health, Institute of Psychology, Beijing, China; ^7^ Department of Psychology, University of Chinese Academy of Sciences, Beijing, China

**Keywords:** treatment-resistant, ziprasidone, sertraline, efficacy, schizophrenia, acute exacerbation

## Abstract

Treatment-resistant schizophrenia (TRS) is a prevalent clinical problem with heterogeneous presentations. However, the clinical trial designs for new treatments are still lacking. This study aimed to assess the efficacy of ziprasidone plus sertraline in TRS patients as compared to ziprasidone monotherapy. We conducted a 24 weeks, randomized, controlled, double-blinded clinical research trial. 62 treatment-resistant patients with acute exacerbation SZ were randomly allocated to receive a usual dose of ziprasidone (120–160 mg/d) monotherapy (Control group) and 53 TRS inpatients were to receive a low dose of ziprasidone (60–80 mg/d) in combination with sertraline (ZS group). Treatment outcomes were measured by the Positive and Negative Syndrome Scale (PANSS), the Hamilton Depression Rating Scale (HAMD), CGI-Severity (CGI-S) and Personal and Social Performance Scale (PSP) at baseline, week 4, 8, 12, and 24. Relative to control group, the patients in ZS group showed greater reductions in the following: PANSS positive symptom, negative symptom, total score, and HAMD total score. Additionally, the patients in ZS group had a greater increase in PSP total score. Notably, the reduction in HAMD was positively correlated with the reduction in PANSS total score. The reduction in CGI-S was a predictor for the improvement of psychosocial functioning in patients. Furthermore, the ZS group had a lower rate of side effects compared to the control group. Our findings suggest that a low dose of ziprasidone in combination with sertraline is an effective therapy for the clinical symptoms as compared to a usual dose of ziprasidone in the treatment-resistant patients with acute exacerbation SZ.

**Clinical Trial Registration:** ClinicalTrials.gov, identifier NCT04076371.

## Introduction

Antipsychotic drugs are widely used for treating patients with schizophrenia (SZ) (Cheng et al., 2019). However, the antipsychotic efficacy is not always satisfactory. For patients who are unresponsive/under-responsive to antipsychotic drugs, treatment remains a major challenge. Studies report that approximately 20%–30% of patients with SZ respond poorly or even insufficiently to antipsychotics, resulting in treatment-resistance schizophrenia (TRS) (Lehman et al., 2004; Howes et al., 2017). The psychiatric symptoms of TRS patients cannot be effectively alleviated, which leads to an increase in morbidity and mortality. Clearly, the treatment of TRS patients continues to be challenging in the clinical management.

Clozapine has been the only recommended drug therapy for refractory SZ for the past 30 years (Kane et al., 1988). Although several randomized controlled clinical trials have demonstrated that clozapine is effective in reducing the symptoms and hospitalizations of TRS (Lewis et al., 2006; McEvoy et al., 2006), the efficacy of clozapine on TRS patients still varies amongst each patient. Specifically, a network meta-analysis of randomized controlled trials (RCT) in TRS patients to compare the efficacy between clozapine and other antipsychotics has reported that clozapine is not superior to most other atypical antipsychotics (Samara et al., 2016). More recently, another meta-analysis has shown that clozapine is effective for the positive symptoms and total symptoms of SZ, regardless of whether or not the patient exhibits resistance (Mizuno et al., 2020). These studies demonstrate that the action of clozapine is not exclusive to the neurobiology underlying treatment-resistance, but instead applies to SZ in general (Mizuno et al., 2020). These findings challenge clozapine’s unique position in the therapeutic effect for TRS patients. Even for patients with good efficacy of clozapine, the various and severe side effects of clozapine and monitoring requirements, including sedation, hypersalivation, postural hypotension, dysphagia, gastrointestinal hypomotility, weight gain, diabetes mellitus and dyslipidaemia, agranulocytosis, cardiomyopathy, myocarditis, pneumonia and seizure, have curtailed its wide adoption and use (Flanagan et al., 2020). Altogether, all these findings demonstrate that exploring effective treatment strategies other than clozapine, including antipsychotics monotherapy or combination of several atypical antipsychotics, presents a shared challenge for psychiatrists globally.

The superiority of several antipsychotics other than clozapine has been evaluated in the treatment TRS patients. For example, a 14 weeks RCT study found that compared to haloperidol, both olanzapine and clozapine have a modest effect on the symptoms of patients with TRS (Volavka et al., 2002). In contrast, another comparative trial demonstrated that olanzapine has no advantageous efficacy towards the symptoms of TRS patients when compared with chlorpromazine in an 8 weeks RCT study (Conley et al., 1998). Notably, in several longitudinal studies, ziprasidone showed greater improvement in negative symptoms of TRS than chlorpromazine in both a 12 weeks and a one-year follow-up study (Kane et al., 2006; Loebel et al., 2007). This indicates the potential clinical efficacy of ziprasidone for the treatment of the psychiatric symptoms in TRS patients. However, the clinical application of ziprasidone is limited because it carries a risk of fatal arrhythmias by prolonging the QTc interval by more than 60 ms above baseline, causing torsades de pointes (TdP) and even sudden cardiac death (Viskin 1999). In addition, to alleviate the side effects of ziprasidone, this study adopted a low dose ziprasidone plus sertraline to explore a clinical pharmacological treatment with few clinical side effects and comparable efficacy to standard dose of ziprasidone.

Recent studies found that patients with TRS may be relatively distinct from patients with treatment-responsive SZ (Gillespie et al., 2017). TRS may be a group of independent subtypes of SZ, rather than just more severe SZ. Numerous studies show that the differences between TRS and treatment-responsive SZ may be attributable to inherently different underlying physiological mechanisms (Demjaha et al., 2014). Recent hypotheses for the pathogenesis of TRS have focused on the abnormal functioning of dopaminergic pathways, changes in glutamate and 5-HT, or changes in several other neurotransmitter pathways. It is speculated that a combination of physiological pathways converges and contributes to the neurobiology of TRS (Potkin et al., 2020). Sertraline is a selective serotonin reuptake inhibitor (Doogan and Caillard 1988). Serotonin plays a complex role in modification of dopaminergic neurotransmission and is thought to have some efficiency in improving the depressive symptoms of SZ as augmentation therapy in combination with antipsychotics medications. Administration of sertraline is more effective than a placebo towards improving postpsychotic depressive symptoms in stable chronic patients with SZ (Mulholland et al., 2003). Therefore, in this study, serotonin was used to alleviate the depressive symptoms in TRS patients.

To investigate whether ziprasidone at a low dose (60–80 mg/d) in combination with sertraline is effective in the treatment of treatment-resistant patients with acute exacerbation SZ, we conducted a 24 weeks, double-blind, and randomized trial in TRS patients. We hypothesized that there would be a significant improvement in the acutely relapsed patients treated with ziprasidone in combination with sertraline relative to ziprasidone monotherapy (120–160 mg/d). Additionally, when considering the close relationship between improvement of clinical symptoms, depressive symptoms, and psychosocial functioning, we hypothesized that treatment-resistant patients with acute exacerbation SZ treated with ziprasidone in combination with sertraline would show greater improvement in personal and psychosocial functioning as measured by Personal and Social Performance Scale (PSP) scale when compared to the control group.

## Methods

### Participants

We conducted the study from January 2014 to June 2015. Participants were recruited from patients in First Hospital of Shanxi Medical University. The study protocol was approved by the Institutional Review Board of First Hospital of Shanxi Medical University. Each participant provided informed consent and signed the written informed consent form.

We recruited 128 treatment-resistant patients with acute exacerbation SZ that were all diagnosed with SZ as determined by the Structured Clinical Interview for DSM-IV (SCID). Acutely relapsed patients were required to meet the following inclusion criteria: 1) antipsychotic treatment (at least two chemical classes) with a dose equivalence of chlorpromazine ≥800 mg/day for 6 weeks, each without significant relief of clinical symptoms, and failure to improve by at least 20% in total BPRS score; 2) a Brief Psychiatric Rating Scale (BPRS) score ≥45 and a Clinical Global Impressions Severity scale (CGI-S) score ≥4 in this evaluation; 3) no stable good social and/or professional functioning in the last 5 years. Patients that met any of the following exclusion criteria were not admitted to the study: 1) abuse or substance dependence except tobacco; 2) with a history of prolonged QT interval; 4) with severe arrhythmia, sinus bradycardia and elevated myocardial enzymes; 5) with self-injury or destructive and unprovoked violence or suicide; 6) with comorbid somatic diseases or abnormal values of routine biochemical tests caused by side effects. We obtained the medical history, electrocardiogram detection, and physical examination from all patients to exclude any patients with a somatic disorder.

### Intervention

We conducted a 24 weeks, randomized and controlled clinical trial. A computer was used to generate the randomization numbers. Subsequently, an independent third-party randomly divided the participants into two groups based on the assigned computer-generated randomization number. Both scale-raters and patients were blinded to the treatment allocation. Treatment allocation was exclusively known by a single nurse who did not participate in this study.

The study group comprised 53 patients who received capsulized ziprasidone (60–80 mg/day) in combination with sertraline 50 mg/day (ZS group). The control group comprised 62 patients that received identically capsulized ziprasidone (120–160 mg/day) monotherapy (Control group). The dose of ziprasidone in both groups was flexible to comport with doctors’ recommendations. The sertraline dose was held constant at 50 mg daily. No adjustment of the types of antipsychotics was allowed during the clinical trial to maintain consistency. Inpatients taking other type of antipsychotics were permitted to receive overlapping antipsychotic therapy within 7 days before entering the study to gradually transition them to the study medication. Moreover, patients in both groups were given 2–3 mg/d Clonazepam *per orem* during the first 4–6 weeks of treatment to improve sleep and reduce both anxiety and acute agitation symptom as soon as possible.

### Objectives and Outcomes

Aggregate psychiatric symptoms as evaluated by the Positive and Negative Syndrome Scale (PANSS) and CGI-Severity (CGI-S) were the primary outcome measure of this study (Guy 1976; Kay et al., 1987). In this study, a 5-factor model was also used to capture the validity of the clinical symptom dimension, which consists of five factors, namely, positive, negative, disorganization, excitement, and depression factors (Wallwork et al., 2012). Secondary outcome measures were both depressive symptoms, as evaluated by the Hamilton Depression Rating Scale (HAMD) (Hamilton 1960), and social functioning, as evaluated by Personal and Social Performance Scale (PSP) (Morosini et al., 2000). Psychiatric symptom and depressive symptom were evaluated at the following time points: baseline; end of week 4, end of week 8, end of week 12; and end of week 24. The raters received a training course before the clinical trial began. After training, the inter-observer correlation coefficients for HAMD total score and PANSS total score were maintained at >0.8 during repeated assessments.

### Assessment of Adverse Events

All patients underwent electrocardiogram monitoring for cardiac side effects, checking for any significant arrhythmias or other serious abnormalities at baseline, at the end of weeks 4, 8, 12, and 24. In addition, blood samples were collected at 7 AM following overnight fasting. Serum levels of Creatine Kinase (CK), creatine kinase-MB (CK-MB), hydroxybutyrate dehydrogenase (HBD), and lactate dehydrogenase (LDH) were all measured in the hospital laboratory center using commercially available kits. Moreover, we used the Treatment Emergent Symptom Scale (TESS) to evaluate adverse treatment effects (Zhang 1984).

### Statistical Analysis

Demographic characteristics, baseline psychiatric symptoms, depressive symptoms, and social functioning were compared between the two groups using analysis of variance (ANOVA) or the chi-square test.

To compare the efficacy in the two trial groups, the repeated measures multivariate analyses of variance were performed as the main statistical strategy. The last observation carried forward (LOCF) analysis was performed on patients who dropped out after treatment. All data were analyzed using an intention to treat design (ITT). After the repeated measures ANOVA, the significant multivariate omnibus tests were followed with a test of individual univariate effect. For the dependent variables, 5 time points (baseline, week 4, week 8, week 12 and week 24) were used as the repeated measures within-effect, and group (monotherapy vs. combination) was used as the between-effect. In each repeated measures ANOVA model, the independent variables were PANSS scores, HAMD scores, CGI scores, and PSP scores over time, respectively. If the group × time interactions were significant, then the group differences at week 4, week 8, at week 12 and week 24 were respectively analyzed by ANCOVA with the baseline score as a covariate. Regression analysis was used to assess the significant factors that correlated with the changes in the outcomes. The following factors associated with the changes in outcomes were included in the regression analysis as independent variables: PANSS changes, age, sex, education years and illness duration. If the group × time interaction effect was not significant, further statistical testing was not performed.

To adjust for multiple testing, Bonferroni corrections were applied. All statistical analyses were conducted in PASW Statistics, version 22.0 (SPSS, Inc., Chicago). The threshold for significance was set at *p* < 0.05.

## Results

### Demographic and Basic Descriptive Data

**FIGURE 1:**
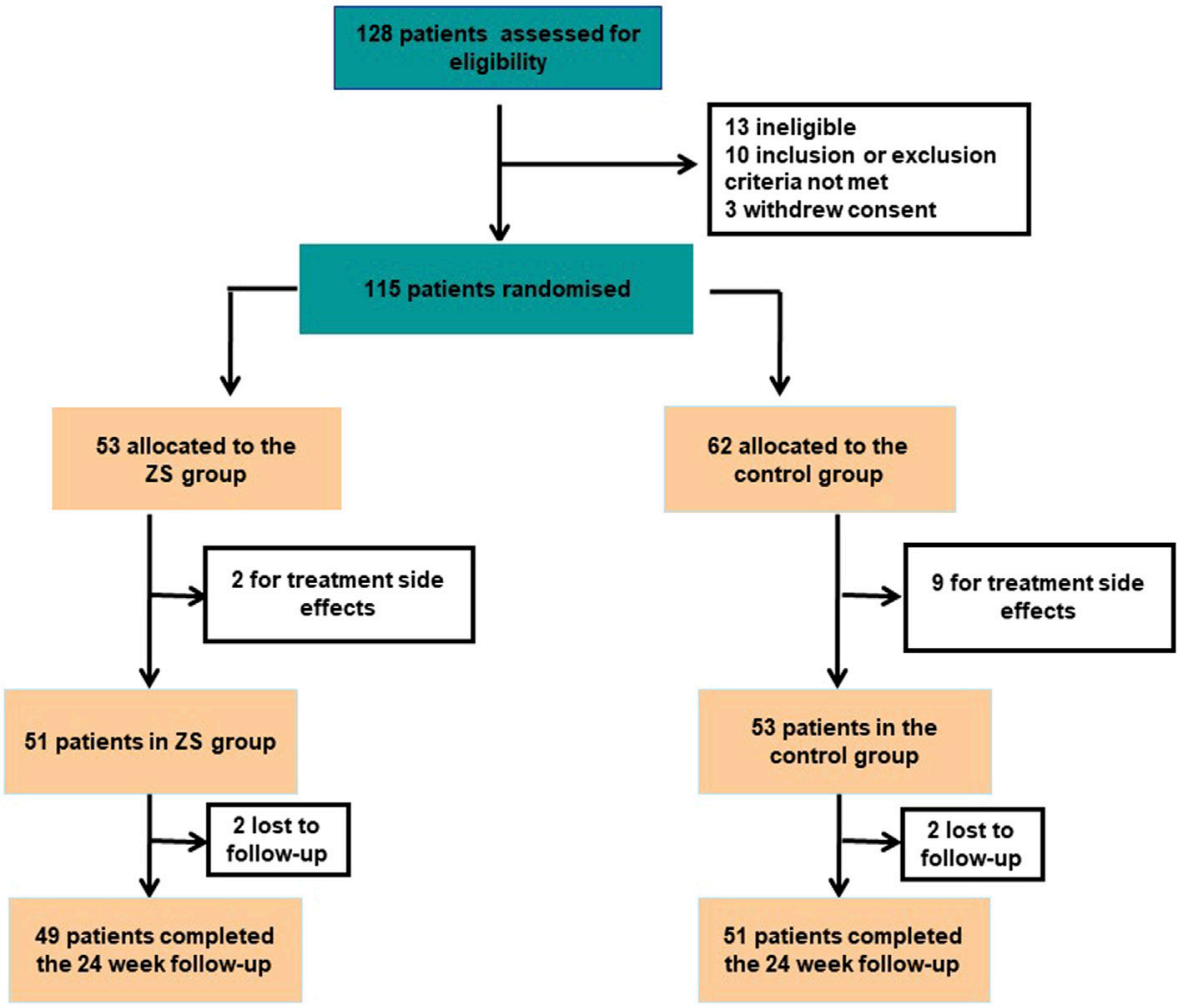
Flow diagram of included studies.

In this study, 4 patients were lost to follow-up by the end of 24 week period due to unanticipated discharge (*n* = 2) and patient decision (*n* = 2) (Figure 1).

At baseline, there were no statistical differences between ZS and control groups in age, education level, sex and onset age (all *p* > 0.05). However, patients in the control group had higher scores in PANSS positive subscore and PSP total score compared to the ZS group, while at the same time, maintaining lower scores in PANSS negative score and CGI-S score (all *p* < 0.05) (Table 1). No significant differences in QTC, CK, CK-MB, HBD and LDH were found between the two groups (all *p* > 0.05). Baseline PANSS total score was correlated with baseline HAMD total score (r = 0.43, *p* < 0.001) and PSP total score (r = −0.33, *p* < 0.001).

**TABLE 1:** Demographic characteristics and clinical data in combination therapy group (ZS) and control group (mean ± standard deviations).

Variable	ZS group (*n* = 49)	Control group (*n* = 51)	F Or *X* ^2^ (*p* value)
Gender (male/female)	31/18	25/26	2.1 (0.15)
Age (years)	41.1 ± 5.0	41.5 ± 6.8	0.1 (0.76)
Education (years)	11.5 ± 2.3	11.3 ± 2.5	0.2 (0.69)
Lab results, mean ± SD			
QTC	393.0 ± 9.2	393.1 ± 10.2	0.002 (0.97)
HR	82.7 ± 6.5	82.3 ± 5.5	0.1 (0.79)
CK	86.2 ± 29.0	87.7 ± 35.6	0.1 (0.81)
CK-MB	8.1 ± 3.2	8.1 ± 3.3	0.001 (0.97)
LDH	158.0 ± 26.4	161.0 ± 29.4	0.3 (0.59)
HBD	124.2 ± 17.0	125.3 ± 16.0	0.1 (0.75)
Age of onset (years)	27.3 ± 4.2	28.5 ± 5.0	1.8 (0.18)
Duration of illness (months)	14.0 ± 4.9	13.1 ± 5.1	0.9 (0.35
Clinical symptoms, mean ± SD			
PANSS *p*	21.9 ± 5.5	25.5 ± 8.0	6.8 (0.01)
PANSS N	34.9 ± 7.8	31.1 ± 9.5	4.7 (0.03)
PANSS G	64.3 ± 9.3	63.2 ± 9.1	0.4 (0.54)
PANSS total score	121.0 ± 12.7	119.7 ± 12.0	0.3 (0.60)
CGI-S score	6.3 ± 0.6	5.9 ± 0.8	7.4 (0.008)
PSP	29.3 ± 4.9	31.9 ± 5.6	6.3 (0.01)
HAMD	27.2 ± 4.2	26.4 ± 5.0	0.8 (0.37)
HAMA	28.4 ± 5.8	28.1 ± 5.7	0.05 (0.83)

Note: CK, creatine kinase; CK-MB, creatine kinase-MB; HBD, hydroxybutyrate dehydrogenase; LDH, lactate dehydrogenase; SD, standard deviation, *p* positive subscore, N negative subscore, G general psychopathology subscore; HAMD, hamilton depression rating scale; PSP, personal and socialperformance scale; CGI-S, Clinical Global Impression-severity.

### Primary Outcomes

**FIGURE 2:**
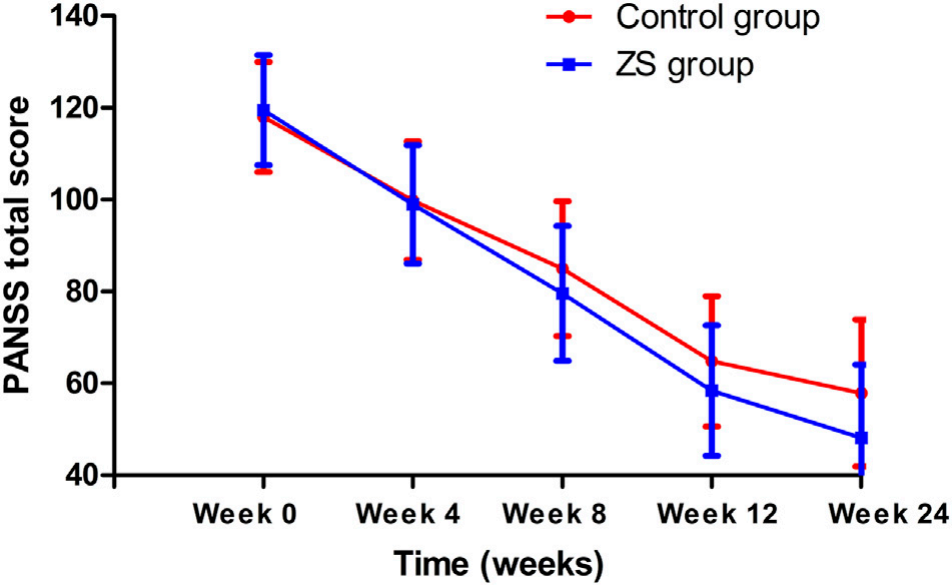
The trajectory of the PANSS total scores in each treatment group.

We evaluated whether the combination therapy significantly improved the clinical symptoms as compared to the control group. Repeated measured ANOVA with PANSS score and CGI-S score as outcome measures revealed a group × time interaction (Wilks’ lambda F = 7.1, *p* < 0.001) and a main effect for the time (Wilks’ lambda F = 11.4, *p* < 0.001). Univariate analyses showed significant group-by-time effects on negative subscore (F = 24.7, *p* < 0.001), PANSS total score (F = 4.0, *p* = 0.04) and CGI-S (F = 5.0, *p* = 0.001) (Figure 2). Significantly, we found notable group effects on positive subscore (F = 5.1, *p* = 0.03) and total score (F = 5.6, *p* = 0.02) and pronounced time effects on PANSS all subscores and CGI (all *p* < 0.01) (Table 2). Further analysis based on PANSS five factors revealed significant group-by-time effects on negative factor (F = 4.2, *p* = 0.002) and depression factor (F = 2.7, *p* = 0.03). Regarding the HAMD score, we found a significant interaction effect of time × group (F = 16.0, *p* < 0.001) with both significant main effects of time (F = 3.6, *p* = 0.007) and group (F = 13.5, *p* < 0.001). Covariates in the analyses include age and sex.

**TABLE 2:** PANSS scores, HAMD score, CGI-S and PSP at baseline, week 4, week 8, week 12 and week 24 in ziprasidone in combination with sertraline (ZS) group and ziprasidone monotherapy (control) group (mean ± standard deviations).

	Baseline (*n* = 115)	Week 4 (*n* = 115)	Week 8 (*n* = 115)	Week 12 (*n* = 115)	Week 24 (*n* = 115)	Group×Time F(*p* value)^a^
PANSS positive score						0.6 (0.49)
ZS group	21.5 ± 5.5	15.6 ± 4.2	12.0 ± 3.4	9.7 ± 2.4	8.8 ± 2.4	
Control group	24.1 ± 7.9	18.0 ± 6.5	14.1 ± 5.7	10.9 ± 3.9	10.1 ± 3.8	
PANSS negative score						24.7 (<0.001)
ZS group	34.2 ± 7.9	29.8 ± 7.3	24.7 ± 6.6	20.6 ± 5.9	14.0 ± 4.8	
Control group	31.6 ± 9.0	28.9 ± 9.3	25.9 ± 9.3	22.8 ± 9.2	19.4 ± 9.4	
PANSS general psychological score						2.0 (0.09)
ZS group	63.9 ± 8.9	53.4 ± 8.6	42.8 ± 10.3	28.0 ± 9.3	25.3 ± 10.0	
Control group	62.3 ± 8.9	52.9 ± 9.2	44.9 ± 11.3	31.0 ± 13.3	28.5 ± 14.5	
PANSS total score						4.0 (0.04)
ZS group	119.5 ± 12.0	99.0 ± 12.9	79.6 ± 14.7	58.4 ± 14.2	48.1 ± 16.0	
Control group	118.0 ± 12.3	99.8 ± 13.8	85.0 ± 17.9	64.8 ± 22.8	57.9 ± 25.6	
Positive factor						0.2 (0.91)
ZS group	13.8 ± 3.1	11.6 ± 4.5	9.9 ± 4.3	7.9 ± 4.0	6.7 ± 3.4	
Control group	15.0 ± 3.9	13.0 ± 5.4	11.4 ± 4.6	9.7 ± 4.3	8.3 ± 4.1	
Negative factor						4.2 (0.002)
ZS group	26.6 ± 5.2	24.8 ± 5.8	23.2 ± 5.9	21.0 ± 5.6	16.5 ± 5.3	
Control group	25.7 ± 5.9	25.0 ± 7.9	23.1 ± 7.1	21.8 ± 8.1	17.8 ± 8.1	
Cognitive factor						2.1 (0.08)
ZS group	12.1 ± 3.2	10.5 ± 3.1	9.2 ± 3.1	8.0 ± 2.9	6.5 ± 2.5	
Control group	11.9 ± 2.7	10.7 ± 2.7	9.7 ± 2.8	8.6 ± 3.0	7.1 ± 3.1	
Depression factor						2.7 (0.03)
ZS group	11.6 ± 4.1	10.3 ± 3.8	9.1 ± 3.5	7.9 ± 3.0	6.2 ± 2.7	
Control group	10.8 ± 3.5	9.5 ± 3.1	8.4 ± 2.8	7.3 ± 2.7	6.3 ± 2.6	
Excitement factor						0.3 (0.89)
ZS group	16.7 ± 4.2	14.8 ± 4.0	12.3 ± 3.8	10.3 ± 3.6	8.2 ± 3.7	
Control group	16.8 ± 4.1	14.9 ± 3.7	12.1 ± 3.4	10.2 ± 3.3	8.5 ± 3.3	
CGI-S score						5.0 (0.001)
ZS group	6.2 ± 0.7	5.4 ± 0.5	4.1 ± 0.8	3.7 ± 0.8	3.4 ± 1.1	
Control group	5.9 ± 0.7	5.1 ± 0.6	4.2 ± 0.9	3.8 ± 1.0	3.5 ± 1.4	
HAMD total score						16.0 (<0.001)
ZS group	26.4 ± 4.9	20.6 ± 3.9	13.0 ± 4.1	9.6 ± 4.5	7.2 ± 5.2	
Control group	26.4 ± 4.9	22.1 ± 5.1	17.7 ± 6.2	14.6 ± 6.6	11.7 ± 7.5	
PSP total score						9.1 (<0.001)
ZS group	29.7 ± 5.2	39.4 ± 6.0	57.3 ± 9.1	66.2 ± 10.6	74.0 ± 13.6	
Control group	30.9 ± 5.6	40.6 ± 8.2	53.9 ± 14.2	61.2 ± 17.5	66.6 ± 20.3	

a Adjusted *F* value controlling for sex and age.

Correlation analysis showed that there were significant positive associations between the reductions from baseline in HAMD score and PANSS total score and CGI score (all *p* < 0.05). Further multiple linear regression analysis found that the reduction in PANSS total score was positively associated with the reduction of HAMD (all *p* < 0.05), after controlling for age, sex, education years, duration of illness and onset age.

### Secondary Outcomes

A repeated measured ANOVA with PSP score as the independent variable showed a group × time interaction (F = 9.1, *p* < 0.001) and a main effect for the time (F = 4.1, *p* = 0.003). Further ANCOVA analysis showed that the PSP score was significantly higher in the ZS group than in the control group at 24 weeks follow-up (F = 8.7, *p* = 0.004). Correlation analysis showed that there was a significant positive association between the reduction from baseline in CGI or PANSS total score and PSP scores (all *p* < 0.05).

Further multiple linear regression analysis was performed to elucidate the predictors of the treatment effect in the ZS group. The treatment effect was represented by the increase in PSP from week 24 to baseline. The covariates included sex, illness duration, age, education year, baseline PSP and reduction of PANSS total score, HAMD, and CGI-S. The results showed that the reduction of PANSS total score was a significant predictor of PSP improvement from baseline to week 24 in the ZS group (all *p* < 0.05).

### Treatment Side Effect

11 patients dropped out due to side effects of the medication. 7 patients were QTC interval prolongation >485 (2 in ZS group and 5 in Control group); 2 patients were QTC interval prolongation >485 with heart rate slowing down to 47 centigrade 45 beats/min and syncope (0 in ZS group and 2 in Control group). 2 patients were frequent multiple ventricular premature beats and significant changes in ST-T (0 in ZS group and 2 in Control group).

In addition, we found significant interactive effects of group × time interaction on QTC, HR and CKMB (all *p* < 0.01). We further found that the QTc interval was shorter in ZS group than that in control group after treatment (*p* < 0.001). Moreover, serum HR levels were significantly lower and CKMB levels were higher in control group than those in ZS group (all *p* < 0.01) (Table 3). ZS patients had fewer side effects measured as TESS (Table 4).

**TABLE 3:** QTc and cardiac biomarkers at baseline, week 4, week 8, week 12 and week 24 in ZS group and control group (mean ± standard deviations).

	Baseline (*n* = 115)	Week 4 (*n* = 115)	Week 8 (*n* = 115)	Week 12 (*n* = 115)	Week 24 (*n* = 115)	Group×Time F(*p* value)^a^
QTC						15.9 (<0.001)
ZS group	393.1 ± 8.9	405.9 ± 12.5	413.9 ± 11.1	420.0 ± 11.7	427.7 ± 12.5	
Control group	392.1 ± 8.9	423.0 ± 16.7	430.3 ± 20.8	435.8 ± 23.1	448.5 ± 27.7	
HR						16.6 (<0.001)
ZS group	82.8 ± 6.3	80.3 ± 5.6	75.9 ± 6.8	72.4 ± 7.0	68.5 ± 6.3	
Control group	83.0 ± 5.3	76.4 ± 6.3	70.5 ± 8.0	64.6 ± 10.6	59.5 ± 12.8	
CK						1.8 (0.17)
ZS group	83.9 ± 29.0	94.6 ± 32.0	109.2 ± 38.0	124.0 ± 42.7	155.2 ± 63.0	
Control group	87.1 ± 34.9	97.9 ± 35.4	110.3 ± 36.1	130.6 ± 43.9	172.4 ± 66.0	
CKMB						9.0 (0.003)
ZS group	8.2 ± 3.1	8.7 ± 2.8	11.7 ± 3.6	10.3 ± 3.2	15.0 ± 5.7	
Control group	7.9 ± 3.2	8.7 ± 2.9	14.5 ± 5.3	11.9 ± 4.0	18.0 ± 7.2	
LDH						1.6 (0.17)
ZS group	157.5 ± 26.2	159.8 ± 22.5	181.3 ± 28.4	165.4 ± 23.3	204.7 ± 45.6	
Control group	159.1 ± 28.6	164.7 ± 28.4	177.3 ± 26.2	178.3 ± 31.5	218.1 ± 39.6	
HBD						2.3 (0.06)
ZS group	124.1 ± 16.6	128.9 ± 17.6	141.2 ± 19.1	143.5 ± 21.3	156.9 ± 23.4	
Control group	124.9 ± 15.0	128.1 ± 22.8	133.4 ± 17.3	144.2 ± 20.9	159.1 ± 22.6	

a Adjusted *F* value controlling for sex and age.

**TABLE 4:** Comparison of adverse effects using Treatment Emergent Symptom Scale between ZS group and control group.

Adverse Events	ZS group (*n* = 53) n (%)	Control group (*n* = 62) n (%)	*X* ^2^ (*p*)
Tremor	4 (7.5)	5 (8.0)	0.01 (0.92)
Akathisia	2 (3.8)	3 (4.8)	0.08 (0.78)
Insomnia	2 (3.8)	3 (4.8)	0.08 (0.78)
Somnolence	1 (2.7)	2 (3.2)	0.2 (0.65)
Constipation	1 (1.9)	2 (3.2)	0.2 (0.65)
Nausea	0 (0)	2 (3.2)	1.7 (0.19)
Dizziness	2 (3.8)	4 (6.5)	0.4 (0.52)
Weight gain	0 (0)	1 (1.6)	0.9 (0.35)
Electrocardiographic changes	2 (3.8)	9 (14.5)	3.8 (0.05)
Muscle rigidity	2 (3.8)	5 (8.0)	0.9 (0.34)
dry mouth	0 (0)	3 (4.8)	2.6 (0.11)

## Discussion

The current findings are as follows: 1) after 24 weeks of treatment, a low dose of ziprasidone in combination with sertraline is more effective in clinical symptoms than ziprasidone monotherapy; 2) combination treatment significantly improves the psychosocial functioning compared to ziprasidone alone; and 3) the improvement in psychiatric symptoms is positively associated with the improvement of psychosocial functioning in the ZS group.

To the best of our knowledge, this is the first study to investigate the efficacy of a low dose of ziprasidone (60–80 mg/d) combined with sertraline towards ameliorating the symptoms of treatment-resistant patients with acute exacerbation SZ. Previous clinical trials predominantly focus on the comparison of efficacy between the usual dose of ziprasidone and other antipsychotics-there appear to be no significant differences in effectiveness as compared to other atypical antipsychotics (Leucht et al., 2013; Huhn et al., 2019). In the present study, we found that the combined treatment was indeed superior to the usual dose of ziprasidone monotherapy in both psychiatric and depressive symptoms. Moreover, the reduction in HAMD score was positively associated with the reductions in PANSS score. One particular strength in our study results from the fact that our patient population is that of an inpatient cohort, which by nature of their hospitalization, helps reduce the rate of treatment noncompliance. Because the patients stayed in the hospital throughout the trial, the nurses in the ward were able to closely monitor their adherence to the antipsychotic medications.

Recent meta-analyses have found the effect of antidepressants in addition to antipsychotics in SZ, mostly with large to medium effect sizes (Helfer et al., 2016; Correll et al., 2017; Gregory et al., 2017; Galling et al., 2018). One possible explanation for the efficacy of combination treatment on treatment-resistant patients with acute exacerbation SZ is that sertraline shows a regulation effect on the 5-HT system. Several lines of studies demonstrate that compared with treatment-responsive patients, TRS patients’ symptoms may be related to different risk factors, etiology, and pathophysiology (Demjaha et al., 2014). In addition to the dopamine system, abnormalities in the 5-HT transmitter system are also involved in the pathological mechanisms of TRS. Clozapine may exert its antipsychotic effects through binding to the 5-HT receptor subtypes (Ashby et al., 1989). Recent studies show that the 5-HT receptor plays a role in the clinical response to clozapine in patients (Rammes et al., 2004). Ziprasidone is an atypical antipsychotic drug which exerts a combined 5-HT and dopamine receptor antagonist effects (Seeger et al., 1995). Additionally, ziprasidone causes the least amount of weight gain and metabolic complications compared to other atypical antipsychotics (Park et al., 2013). Previous clinical trials support that treatment by typical dose of ziprasidone is effective for over 1 year of maintenance therapy for patients with SZ and is accompanied by low rates of both weight gain and metabolic abnormalities (Loebel et al., 2007). Sertraline is well known to be an antidepressant with potent and specific inhibition of the 5-HT system (Doogan and Caillard 1988). Sertraline can also help treat emotional symptoms in patients with SZ. Indeed, we found a significant reduction in HAMD total score of patients after treatment in the ZS group. Sertraline also acts as a dopamine antagonist to enhance dopaminergic function by blocking DA transporters and reuptake in the treatment of psychiatric symptoms (Richelson 1994; Popli et al., 1997). Moreover, sertraline may bind to sigma-1 receptors and treats psychiatric symptoms in patients with depressive disorder (Albayrak and Hashimoto 2017). The pharmacological mechanisms of sertraline provide the basis for its use in combination with ziprasidone in the treatment-resistant patients with acute exacerbation SZ.

Notably, we used a low-dose ziprasidone to treat the treatment-resistant patients with acute exacerbation SZ in this trial. We found that the adverse event rate in combination treatment was lower than ziprasidone monotherapy, which has important clinical implications for the treatment and use of ziprasidone. Compared with other antipsychotics, ziprasidone is superior with regards to the following side effects: incidences of extrapyramidal adverse effects, effects on serum levels of glucose, prolactin, lipids, and weight (Bouchette et al., 2021). However, ziprasidone is known to increases the rate of sudden cardiac death related to the dose-dependent prolongation of the QTc interval. Ziprasidone is also linked to fatal arrhythmias such as apical torsion ventricular tachycardia in patients, thus limiting its widespread use in clinical practice. Our finding was consistent with a previous 6 weeks placebo-controlled trial by Daniel et al., which reported that low-dose ziprasidone (80 mg/d) could significantly improve the PANSS total, CGI-S, and PANSS negative scores in patients with an acute exacerbation of SZ or schizoaffective disorder (Daniel et al., 1999). A noticeable difference between our study and Daniel et al. was that the patients were different (patients with SZ vs. TRS). Also, in the Daniel study, patients took short-term antipsychotics (6 weeks), while in our study, patients were treated with antipsychotic drugs over a medium-term (24 weeks). Some studies show that antipsychotic polypharmacy/combination treatment usually increases the total dose of antipsychotics, increases the interaction of drugs, and increases the risk of antipsychotics-related side effects. Our study was remarkable in the sense that the results showed a lower risk of side effects in patients in combination treatment group as compared to the control group. We found no statistically significant difference in the rate of side effect between the two groups. In the aggregate, our findings suggest that a low dose of ziprasidone and sertraline combination therapy is an effective and safe treatment option for treatment-resistant patients with acute exacerbation SZ.

Surprisingly, we found that combination therapy significantly improved the psychosocial functioning of treatment-resistant patients with acute exacerbation SZ as indicated by the increase in PSP total score. 80% of patents with SZ have persistent social dysfunction. Accumulating studies have realized the importance of psychosocial functioning improvement in patients with SZ and considered both clinical remission and social function recovery in treatment plans (Vita and Barlati 2018). A previous clinical trial reported that functioning recovery is difficult for patients with SZ (Velthorst et al., 2017), which is impaired in the initial stage of the illness and persistent during the course of the disease. Hence, our findings of social functioning improvement as measured in PSP score have significant clinical implications. Notably, we also found that the reductions in broad measures of disease severity including PANSS and CGI-S were positively associated with the increase in PSP. When a cut-off value was set in the improvement of ≥6 points in PSP score, we also found a significant association between the improvements in CGI-S and PSP. These results were similar to previous prospective studies (Vauth et al., 2020), suggesting that combination therapy might play a role by improving symptoms, increasing patient enthusiasm, enhancing patient communication ability, and increased participation in social activities. Previous studies also found that the psychiatric symptoms-like negative symptoms-were a predictor for social functioning (Best et al., 2020). Despite this, we did not find a positive association between the increase in PSP and reduction of HAMD. This lack of positive association demonstrates that improvement of psychiatric symptoms-rather than improvement of depressive symptoms-play a critical role in patient outcomes. These assessed variables account for 29% of the variance in psychosocial functioning improvement in the linear regression model. From a clinical perspective, only a small component of variation in the PSP is explained by the improvement of psychiatric symptoms. However, there are other relevant factors that should be afforded attention in future studies.

This study has several limitations. First, our findings are limited by a relatively small sample size that may bias the data pool-a subsequent trial with a larger size sample is therefore warranted. Second, considering the relapse of patients with SZ in part results from medical non-adherence or discontinuation, it is still hard to distinguish true TRS from pseudo-resistance among the recruited patients. Challenging inadequate rather than ineffective treatment can cause a patient to appear resistant to antipsychotics. Third, the main limitation is that this was not a placebo-controlled, double-blinded clinical trial. Although patients were not told what drugs they were taking, and patients were not mutually aware of the number of capsules taken during the trial, patients were able to recognize the intervention because those who were assigned to the ZS group needed to take 2 capsules while those who were assigned the control group needed to take 1 capsule. Fourth, we did not collect detailed doses of ziprasidone in each treatment group in this study.

In conclusion, the results showed that combination therapy was effective for the psychiatric symptom, depressive symptom, and social functioning. The improvement of psychotic symptoms was correlated with the improvement of depressive symptoms. The reduction in psychotic symptoms was also a significant predictor for the improvement of social functioning. Our findings provide a novel treatment option for treatment-resistant patients with acute exacerbation SZ that may improve efficacy while simultaneously minimizing deleterious effects from antipsychotic drugs. Although the results of this study are encouraging, their replication in a larger patient population is necessary-we remain optimistic in the interim. In particular, clozapine has been used in the treatment of TRS patients, however, this study did not compare the efficacy of the combination of low dose of ziprasidone and sertraline with clozapine, which will need to be tested in a future clinical trial.

## Data Availability

The raw data supporting the conclusion of this article will be made available by the authors, without undue reservation.
